# Turning Image Sensors into Position and Time Sensitive Quantitative Colorimetric Data Sources with the Aid of Novel Image Processing/Analysis Software

**DOI:** 10.3390/s20226418

**Published:** 2020-11-10

**Authors:** Yeongsik Yoo, Woo Sik Yoo

**Affiliations:** 1 College of Liberal Arts, Dankook University, Yongin-si 16890, Gyeonggi-do, Korea; ysyoophd@dankook.ac.kr; 2 WaferMasters, Dublin, CA 94568, USA

**Keywords:** still image, video image, image analysis, colorimetry, software development

## Abstract

Still images and video images acquired from image sensors are very valuable sources of information. From still images, position-sensitive, quantitative intensity, or colorimetric information can be obtained. Video images made of a time series of still images can provide time-dependent, quantitative intensity, or colorimetric information in addition to the position-sensitive information from a single still image. With the aid of novel image processing/analysis software, extraction of position- and time-sensitive quantitative colorimetric information was demonstrated from still image and video images of litmus test strips for pH tests of solutions. Visual inspection of the color change in the litmus test strips after chemical reaction with chemical solutions is typically exercised. Visual comparison of the color of the test solution with a standard color chart provides an approximate pH value to the nearest whole number. Accurate colorimetric quantification and dynamic analysis of chemical properties from captured still images and recorded video images of test solutions using novel image processing/analysis software are proposed with experimental feasibility study results towards value-added image sensor applications. Position- and time-sensitive quantitative colorimetric measurements and analysis examples are demonstrated.

## 1. Introduction

Color is considered a property of objects. Verbal description or written statement of colors is very subjective and often causes miscommunication and/or misunderstanding. We mostly rely, for color judgement and description, on our experience in daily life. In most cases, inaccurate color description does not cause serious problems. It is a matter of preference. However, it can cause unexpected or serious problems including life threatening accidents in medical, pharmaceutical, food science, and agricultural areas. For the fashion industry, paint industry, food industry, commercial product manufacturing industry, and chemical industries we are a little more cautious about color description, because it can affect safety, marketing, and product quality control, and finally, their revenue. The ability to quantitatively determine colorimetric data can provide competitive advantages. Image sensors and spectrometers are essential for these applications [1,2,3,4,5,6]. We would like to introduce the effective use of image sensors as position- and time-sensitive quantitative colorimetric characterization devices with the aid of novel image processing/analysis software. Application examples are given using simple litmus test strips with a wide range of colors depending on the pH value of solutions.

For pH (power of hydrogen or potential for hydrogen in the range of 1–14) tests, pH indicators such as litmus paper made from absorbent paper are used to specify how acidic or basic a water-based solution is. Color is used as an index of composition of test solutions. The color of test solutions is compared with a standard color chart to estimate the nature and properties of test solutions, accurate to the nearest whole number. Unlike a ruler for length measurement, we cannot accurately estimate or imagine colors between any two reference colors. For length measurement using a ruler, we can easily estimate it in decimal points. However, color estimation is very difficult, and estimation results can vary significantly between individuals unless colorimetric or spectroscopic quantification techniques are utilized [7,8]. If color is changed with time, it is even more difficult. We are losing a significant amount of valuable information due to our inability for objective and quantitative analysis of color by the naked eye.

For record keeping purposes, we often take photographs and/or video clips. Digital cameras, camcorders, and smart phones can save photographs and video clips in digital formats. They are widely available in daily life. Color analysis of digital photographs and video clips can be very useful for the quantification of colorimetric characteristics. It allows objectivity of color analysis and elimination of human error including variations of individual sensitivity. Analysis of time-dependent color change can also provide very valuable insight into changes in chemical properties and reactions in situ and/or ex situ.

In this paper, colorimetric quantification and dynamic analysis of various chemical properties from captured still images and recorded video images are proposed for accurate and objective characterization of experimental evidence acquired through solid-state image sensor(s) used in digital cameras, camcorders, smart phones, and digital microscopes. For colorimetric quantification and dynamic analysis examples of still and video images under various test and controlled (color and brightness varying) illumination conditions, images of pH tests using litmus paper strips and unreacted litmus test strips under the controlled illumination conditions are analyzed by novel image processing/analysis software (PicMan [9,10,11] from WaferMasters, Inc., Dublin, CA, USA) and proposed future applications.

## 2. Materials and Methods

### 2.1. Litmus Test Strips

**Figure 1:**
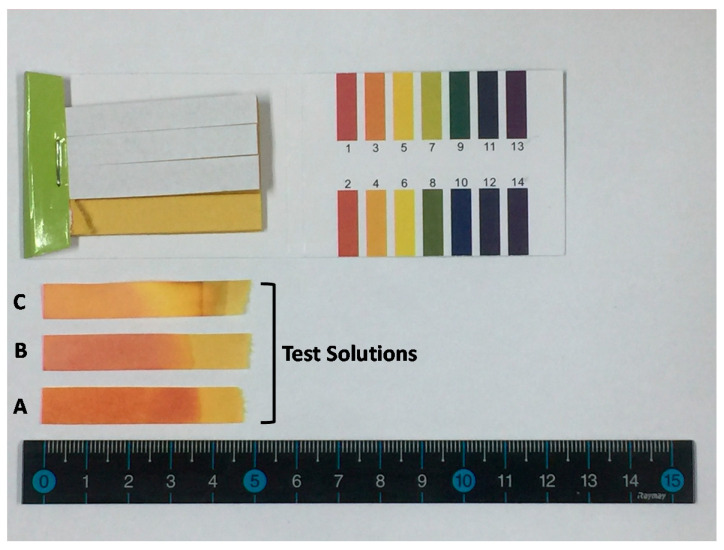
A photograph of litmus test paper strips for the full pH range 1–14 and three strips after test. A: cider, B: refreshment drink, and C: apple vinegar.

A kit for litmus test paper strips is used in the colorimetric study. The litmus paper strips can indicate the full pH range 1–14 (Figure 1). One of the chemical properties of solutions’ pH (power of hydrogen or potential for hydrogen in the range of 1–14) is either tested using pH indicators by visual inspection of color change after chemical reaction with chemical solutions or an electric pH meter. The pH indicators, such as litmus paper made from absorbent paper, change color in accordance with the pH of a test solution. Visual comparison of the color of the test solution with a standard color chart provides an approximate pH value to the nearest whole number. The color chart in the kit shows corresponding color for solutions with pH values in whole number range between 1 and 14. At 25 °C, pH value 7 is neutral. A pH less than 7 is acidic, and solutions with a pH greater than 7 are basic. The neutral value of the pH depends on temperature. It becomes smaller than 7 if the temperature increases. The pH scale is logarithmic and inversely indicates the concentration of hydrogen ions in the solution. A lower pH indicates a higher concentration of hydrogen ions. The pH calculation formula uses the negative of the base 10 logarithm of the molar concentration of hydrogen ions in the solution [12]. The pH value can also be less than 0 for very strong acids, or greater than 14 for very strong bases [13].

Three litmus paper strips tested using three different test solutions A, B, and C from different suppliers are also shown in Figure 1 as real test examples with color variations within tested regions of the strips. Untested litmus paper strips with standard color chart for pH reading were photographed and video recorded under the controlled (color and brightness varying) illumination conditions for time series analysis of color on the standard color chart and white blank area.

### 2.2. Homemade LED Ring Light for Illumination Control

**Figure 2:**
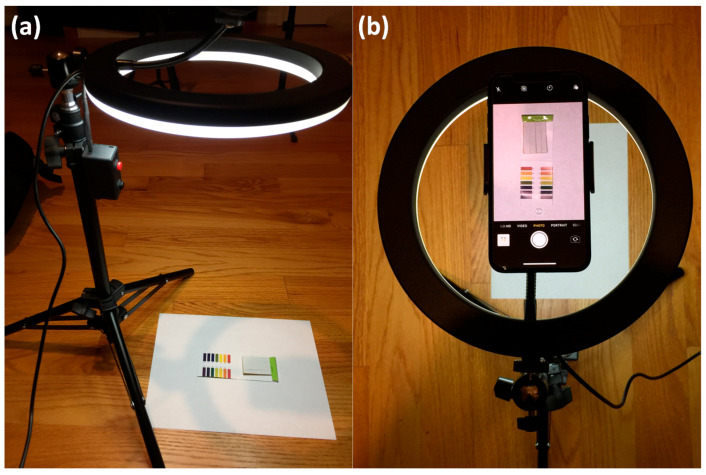
(**a**) Side view of experimental set up (a LED ling light with color code chart of litmus test paper) and (**b**) top view of video recording scene under a controlled LED ring light illumination.

A homemade LED ring light with a smartphone holder fixed on a tripod, capable of illumination color (or hue value) and brightness control, is prepared for investigating the effect of lighting on the appearance of colors on the stand color chart for pH reading. Photographs and video images of the kit for litmus test strips were taken under various illumination conditions. The appearance of the printed standard color chart on white paper was investigated under different lighting (illumination) color (hue value) conditions, as shown in Figure 2.

### 2.3. Image Capturing Devices: USB Digital Microscope and Smartphone

**Figure 3:**
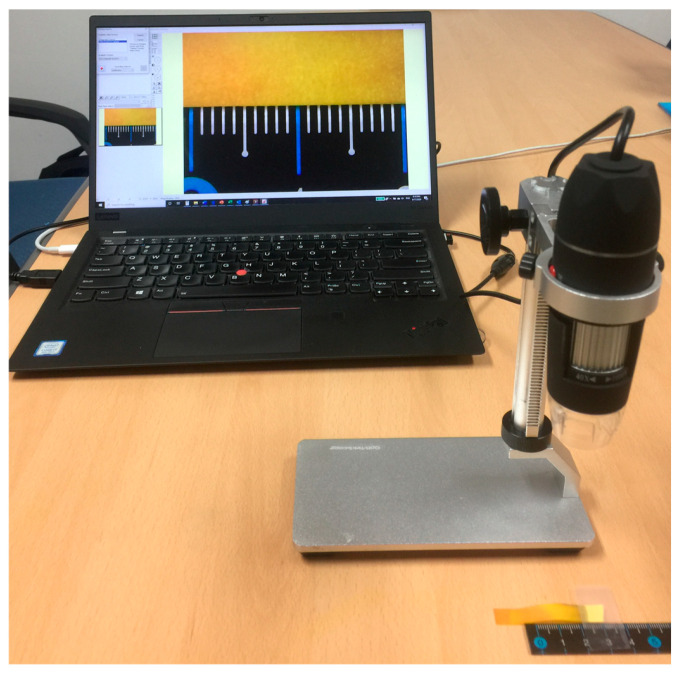
Experimental set up for pH test capable of still/video image recording and colorimetric quantification through image analysis.

A Universal Serial Bus (USB) digital microscope, smartphone, and integrated camera on a personal computer (PC) were mainly used for capturing still images and recording video files. Figure 3 shows one of the experimental set ups for the pH test using litmus test strips. The image on the PC screen is the live image of litmus test paper through the USB digital microscope and the image processing/analysis software (PicMan). The litmus test strip under dynamic pH testing and a ruler on a table are placed under the USB digital microscope for measuring diffusion length and estimating diffusion speed of test solutions on litmus test strips.

### 2.4. Image Processing/Analysis Software: PicMan

For material characterization, various features (shape, size, area, surface area, volume, profile, texture, etc.) of objects of interest must be quantified rather than using visual inspection by human eyes. Visual inspection is very subjective and cannot be used as quantitative data in a reliable manner. A computer-aided decision-making process is highly desired. Quantitative characterization using image processing/analysis software must be implemented for objective analysis and record keeping. For large amounts of image data acquisition, processing, and analysis, automation capability is also a very important feature to have as a useful image processing/analysis software. From these perspectives, a new image processing/analysis software development project was launched by WaferMasters, Inc. several years ago.

A PC with newly developed image processing/analysis software (PicMan [6,7,8,9,10,11] from WaferMasters, Inc., Dublin, CA, USA) capable of the desired features described above and a USB digital microscope were used for capturing still images and recording video images during pH test experiments using three refreshment drinks. Still and video image analysis is done using the image processing/analysis software.

### 2.5. pH Testing Solutions

**Figure 4:**
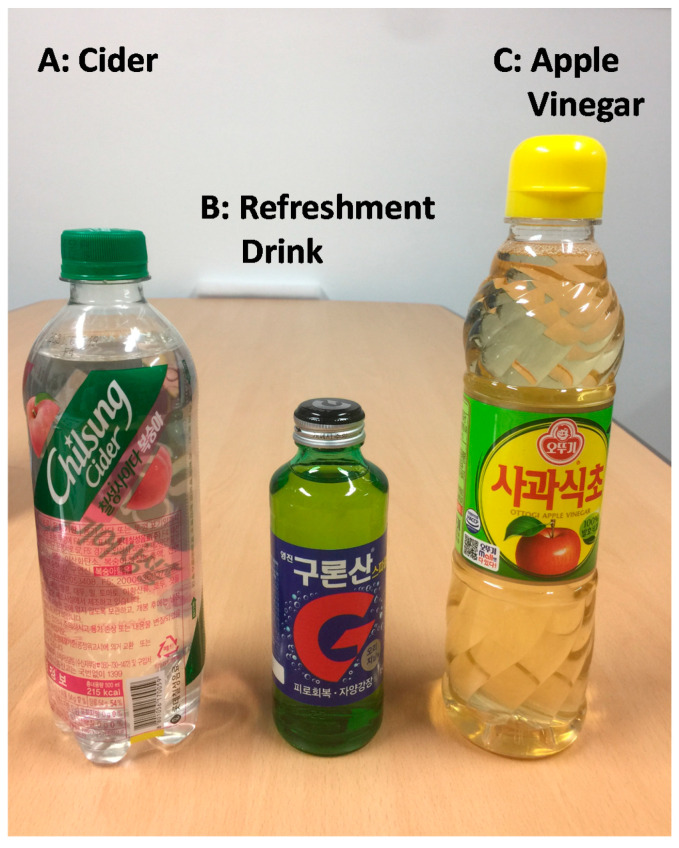
pH test solutions (commercial products in Korea) from different suppliers. A: cider, B: refreshment drink, and C: apple vinegar.

Three different test solutions A (cider), B (refreshment drink), and C (apple vinegar) from different Korean suppliers (Figure 4) were used for demonstrating litmus tests and its photographic and video image analysis.

## 3. Results and Discussion

### 3.1. Still Image Analysis (Static Analysis)

#### 3.1.1. Interpolation of Color Scale

**Figure 5:**
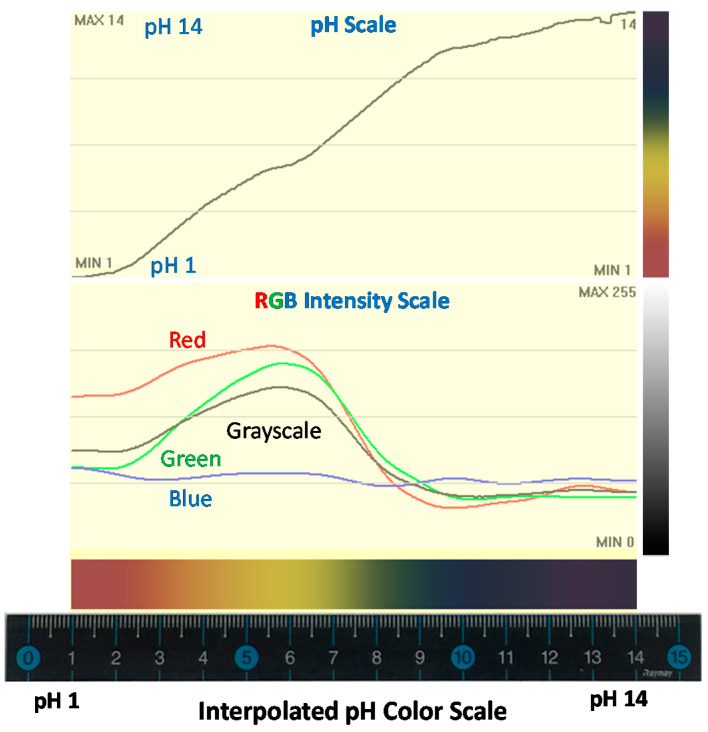
Interpolated pH color scale and its pH conversion linearity (top) with corresponding color component in 8-bit (0–255) red, green, and blue (RGB) brightness.

Litmus test paper strips change color corresponding to the pH values of three test solutions used in this study. The color of tested strips is compared with the reference color chart for pH values from 1 to 14. As seen in Figure 1, there are color variations in individual test strips and between test strips. None of the test strips showed an exact color match with the reference color chart for pH values of whole numbers 1 through 14. It is very difficult to estimate pH values with an accuracy of even ±1 by comparing colors with the naked eye. Considering that the nature of the pH scale is logarithmic, a change in pH value by 1 corresponds to a hydrogen ion concentration change of 10 times. A more accurate and reliable measurement technique needs to be developed. Perhaps, 10 times higher measurement accuracy or 131 reference colors, from 1.0 to 14.0 in 0.1 intervals would be more practical here, instead of 14 reference colors from 1 to 14 in 1 unit intervals, as seen in Figure 5.

In theory, we should be able to generate a continuous color chart using information from 14 discrete reference colors for pH values of 1–14. The discrete color scale for pH values of 1–14 were interpolated to generate a continuous color chart to estimate pH values with 0.1 pH accuracy from the color of test strips (Figure 5). For easy understanding, a 15 cm ruler was placed next to the continuous color scale. The pH values can be directly read from the ruler where the apparent color of tested strips can be viewed.

Each pixel on the continuous color scale represents 24 bits of data (8-bit each for RGB: red, green, and blue). The 8-bit RGB component can have brightness between 0 and 255 (2^8^ = 256 levels). The RGB intensity and its average (grayscale) intensity along the continuous color scale for pH 1.0–14.0 are plotted above the color scale in Figure 5. The color scale was converted to the pH scale for 1.0–14.0 on the top portion of Figure 5. If the color scale is perfectly generated, the slope of the pH scale graph as a function of colors in the range of 1.0 to 14.0 should be a straight line in the graph. As seen from the top portion of Figure 5, the line is not quite straight. However, it covers pH values from 1.0 to 14.0 and provides a reasonable approximation for pH conversion from the actual color of tested strips. The continuous color scale and its conversion formula for pH value estimation can be improved and optimized by collecting more test data and subsequent calibration.

#### 3.1.2. Point Value Reading from Interpolated Color Scale

**Figure 6:**
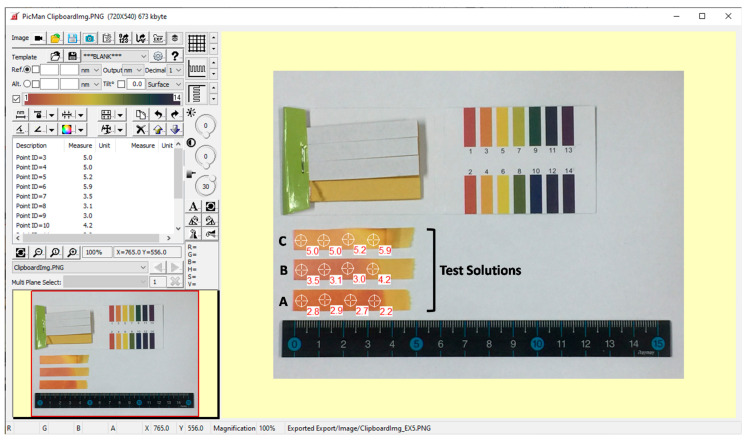
Screen capture image of a noble image processing and analysis software (PicMan) reading pH test values on tested stripes.

The pH values were estimated using the PicMan software from the color information of pixels on the three test strips from test solutions (Figure 6) (Video S1). The continuous color scale for the pH range of 1.0–14.0 is shown at the upper left side of the screen capture image. The pH value on test strip A (cider) spanned from 2.2 to 2.9. Test strip B (refreshment drink) covered the pH range from 3.0 to 4.2. Test strip C (apple vinegar) spanned the pH values between 5.0 and 5.9. Variations in pH values on individual test strips and among test strips were successfully and objectively estimated with pH value of 0.1 divisions without human error. The software was introduced a few years ago and is actively used in material science, semiconductor industry, medical science, cultural heritage, and conservation science with new applications being developed continuously [6,7,8,9,10,11,14].

### 3.2. Video Image Analysis (Dynamic Analysis)

#### 3.2.1. Line Data and Arial Data Extraction

**Figure 7:**
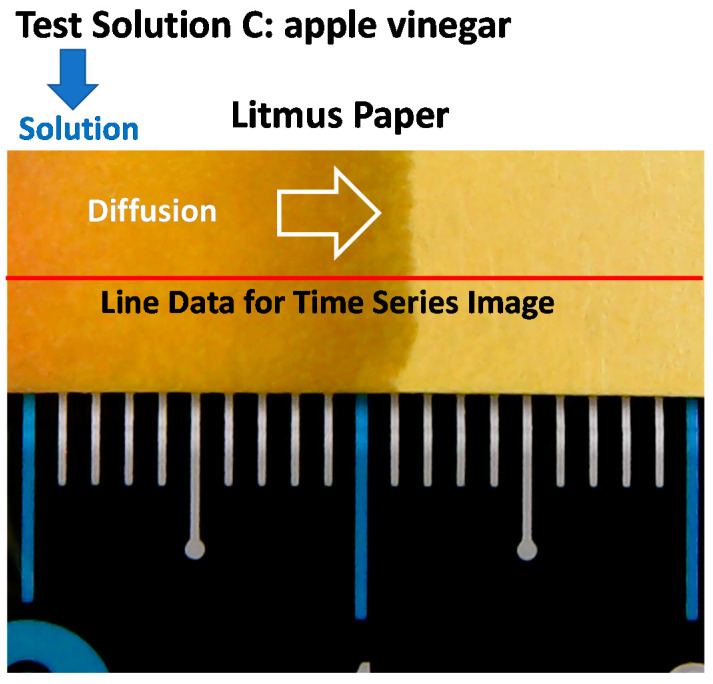
Snapshot image of litmus paper undergoing diffusion of test solution C (apple vinegar). Color information along the red line on the litmus paper is extracted from every frame of video image for dynamic analysis.

Colorimetric information on any subset (scattered points, points in line(s), or points in a selected area) of still images can be extracted and processed and/or rearranged in ways to express information more effectively. Each point in an image is referred to as a pixel. To demonstrate characteristics of the diffusion speed of test solutions on litmus test strips and pH variations with time, video images were recorded and analyzed. A drop of test solution was placed at the edge of a test paper strip, and liquid diffusion and color change were recorded as a video file (Video S2). Figure 7 shows a still image extracted from a video image file 10 s after liquid crossed the left blue tick on the ruler. From the image, we can estimate average diffusion speed of 1.1 mm/s (diffused distance of 11 mm in 10 s) assuming constant diffusion speed. If we can analyze the video images, no assumption is required for diffusion speed characterization.

If we extract colorimetric information along the line perpendicular to the diffusion or reaction front, we will get an exact location of the diffusion boundary and any color variation along the colorimetric sampling line as shown in Figure 7. If we were taking statistics of sampling aerial data, it can provide average, minimum, maximum, range, mode, standard deviation, and more statistical information of colorimetric data on the sampled area.

#### 3.2.2. Line Data Extraction and Image Reconstruction along a Time Axis

**Figure 8:**
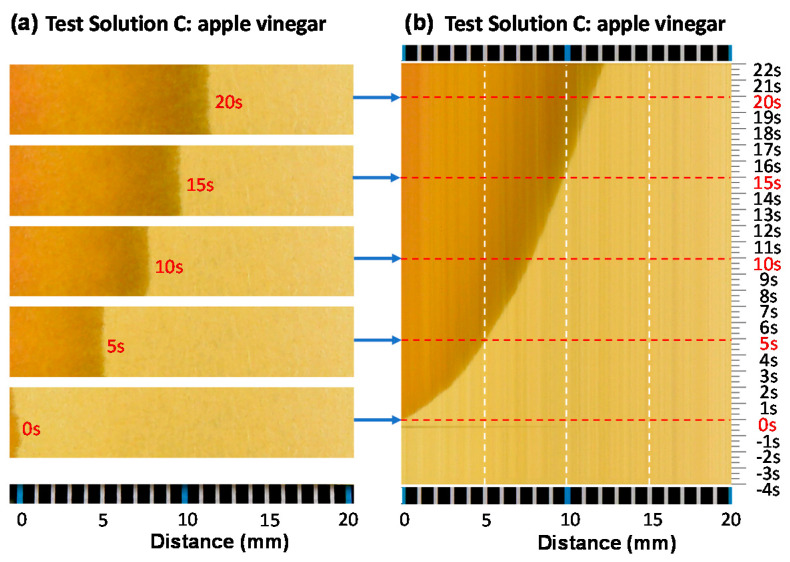
A series of snapshot images (taken at 0, 5, 10, 15, and 20 s) (**a**) and a reconstructed image using color information along center line of every frame (780 still images at 30 fps for 26 s) from −4 to 22 s (test solution C: apple vinegar) (**b**).

Figure 8a shows five still images at 0, 5, 10, 15, and 20 s after the liquid crossed the left blue tick (distance reference: 0 mm). As seen from the figure, the liquid diffusion speed slows down with time. This may be due to the depletion of liquid source and increase in diffusion resistance with diffusion length increase. If we extract color information along the red line (or any line) along the direction of diffusion on the litmus paper from every frame of a video image and reconstruct a time series of images, diffusion dynamics can be visualized as a time cross-section image. No assumption is required for analysis. We recorded video images for 26 s (from −4 s to 22 s) at 30 fps (frames per second). The total of 720 frames of still images (30 fps × 26 s = 720 frames) were recorded in a video file (Video S2). Figure 8b is the reconstructed image from 720 frames. Only one line per frame at the same location (horizontal red line in Figure 7) was extracted and reconstructed as a time series (time cross-sectional) image. As seen from the image, liquid diffusion speed slows with time and showed parabolic or quadratic behaviors. It also shows that the color of the diffusion front is darker, implying a chemical reaction is in progress.

**Figure 9:**
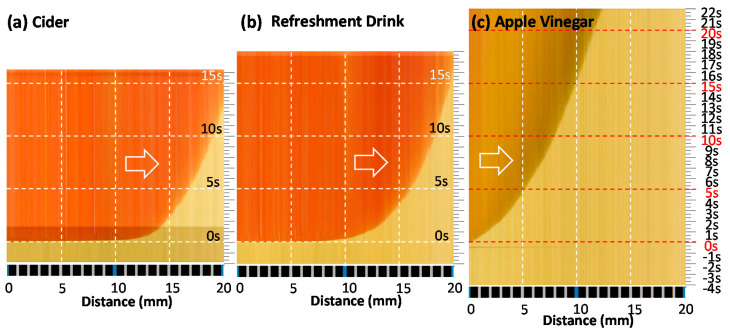
Time series reconstructed images of test solutions for dynamic analyses of diffusion speed and chemical reaction. (**a**) Cider, (**b**) refreshment drink, and (**c**) apple vinegar. Color can be interpreted as pH value, as seen in Figure 6.

**Figure 10:**
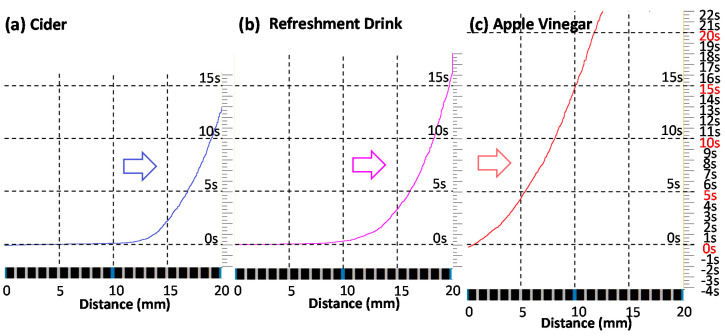
Graphs for diffusion speed and chemical reaction generated from time series reconstructed images of test solutions. (**a**) Cider, (**b**) refreshment drink, and (**c**) apple vinegar.

The diffusion speed of test solutions (A, B, and C) and color change in litmus test paper strips were visualized as time series images by analyzing video files, as shown in Figure 9. It represents the change in color in a selected line on the litmus paper with time. It visualizes the chemical reaction and diffusion on the sampling line on the litmus test strip over time after dropping a drop of test solution C at the edge of the test strip. The colorimetric data in Figure 8 and Figure 9 can be translated into pH values at any point and time. The color image is not only different in appearance but also a rich source of valuable chemical information of test solutions at any point and time. We can even transform the 2D image into a 3D surface plot with distance (x), time (y), and pH (z) axes for more intuitive graphical expression. Figure 10 is the reprocessed image of Figure 9. The location of reaction/diffusion front for every frame was highlighted. It is a simplified image and only carries significantly reduced information (reaction/diffusion front as a function of time with no pH information). It resembles an old-fashioned X–t chart recorder image and an X–Y graph (X axis: distance of diffusion and Y axis: time after test solution drop). We certainly feel much more comfortable with this type of simplified graph, because we were trained that way for a long time. However, there is no reason why we have to intentionally reduce information from the original, information-rich image data. We may need to accept a paradigm shift and find a new way of dealing with information-rich image data without intentional filtering for simplification.

Judging from Figure 10, it is clear that the test solution A (cider) is the fastest diffusing solution through the test strip. The slowest diffusing solution is found to be the test solution C (apple vinegar). The test solution A (cider) and B (refreshment drink) have lower pH values (2.2 to 4.2: strongly acidic) compared to the test solution C (apple vinegar, 5.0–5.9: mild acidity, close to black coffee).

Since these particular video files were recorded at 30 fps, 33.3 ms (1 s/30 fps = 1/30 s) time sensitivity is achieved. High speed (high frame rate) video recording would make more time-sensitive characterization available for fast reaction, fast color change, and/or fast movement. Uniformity or non-uniformity of reaction behaviors can also be quantified through image analysis. Video recording and video file analysis can be very useful for the visualization of time-dependent chemical reactions, such as ink diffusion in water, chromatography, discoloration, etc.

The pH of aqueous solutions can also be electrically measured with a glass electrode and a pH meter. The electrode, or electric pH meter, provide either analog or digital output. It provides one pH value per electrode or meter. The output value is often used for visualization of measurement data in table or graphical form, depending on the applications. In colorimetric characterization techniques, a solid-state (CMOS: complementary metal-semiconductor-oxide or CCD: charge coupled device) image sensor(s) with arrays of pixels are used. Each pixel can carry intensity information on RGB channels and have colorimetric information. Each can be considered as a position-sensitive probe. A still image can be viewed as a two-dimensional (2D) array of colorimetric probes. For video images, three-dimensional (3D: X, Y, and time) sources of information, a video graphic array (VGA) (640 pixel × 480 pixel) image can be viewed as a set of 307,200 pixels (or colorimetric sensors). It can be viewed as a fully integrated, high-density colorimetric sensor array ready for use. Much higher resolution image and video file formats are available. Digital cameras and smartphones with 10 Mega pixel image sensors are not considered high-end anymore. Smartphones with even higher resolution image sensors, up to 16 MP (4920 pixel × 3624 pixel), are readily available. It is more than 50 times better resolution compared to the image sensor for VGA resolution. It is time for turning image sensors into very economic and valuable position- and time-sensitive quantitative colorimetric information generation devices. It does require a paradigm shift. It may be uncomfortable in the beginning, but the benefit will be much greater if we can adapt.

### 3.3. A Case Study of Video Image Analysis: Lighting Effect on Apperance

As briefly described above, video image is a group of snapshot photos in consecutive time sequence with predetermined time spacings (or intervals). When we record video images of a changing object, every frame of video image captures colorimetric information on all pixels in the image sensor and in a sequential order in predetermined intervals (fps: frame per second). Audio information is often recorded together in a synchronized manner. By analyzing video images, we can monitor and quantify the change in color, intensity, shape, and other circumstantial information. Some subtle information such as the mood of a person, facial expressions, etc., cannot easily be identified or classified in an objective manner. However, most apparent features can be quantitatively and objectively characterized for practical applications. Data mining and information extraction from images opens a totally different world for image processing/analysis. It often has to deal with factors of optical illusion and human psychology.

**Figure 11:**
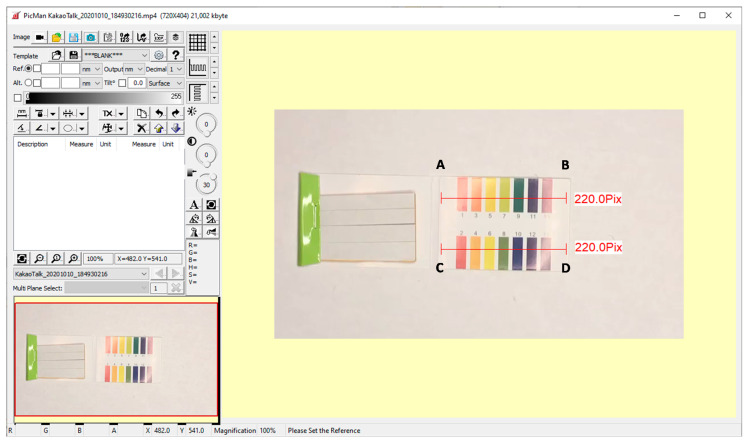
Screen capture image of image processing/analysis software (PicMan) under video image analysis of time resolved color change across the selected cross-sections (A–B and C–D) of color chart printed on a glossy white paper. The video image is recorded under continuously changing lighting conditions.

**Figure 12:**
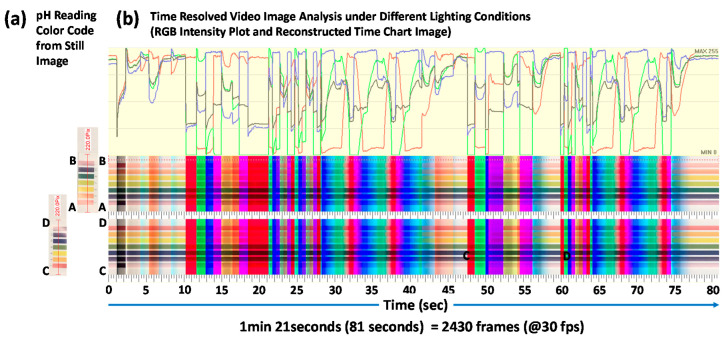
(**a**) pH reading color code look up chart image from a still image and (**b**) time-resolved video image analysis with reconstructed time chart image across A–B and C–D cross-sections of the pH reading color code chart. (RGB intensity graph represents reflected light intensity from the white area of the pH reading color code chart).

Figure 11 shows a screen capture image of image processing/analysis software (PicMan) under video image analysis of time resolved color change across the selected cross-sections (A–B and C–D) of color chart printed on a glossy white paper (Video S3). The video image is 81 s long and was recorded at 30 fps under continuously changing lighting conditions using the controlled LED ring light shown in Figure 2. The video file can be seen from the supplement file. It consists of 2430 frames of still images (81 s × 30 fps = 2430 frames). The selected cross-sections of A–B and C–D have widths of 220 pixels each. The colorimetric information (RGB values) on the cross-sections A–B and C–D of the 2430 frames of still images were reconstructed in Figure 12. Two reconstructed images have 220 pixels × 2430 pixels (width × number of frames) each.

Intensity of RGB channels on a point in the glossy white paper under changing lighting conditions was plotted with red (R), green (G) and blue (B) lines on top of the reconstructed image. The average intensity of RGB values at the point was plotted in gray line. The x axis represents time in the reconstructed image. It is the RGB and average intensity plot as a function of time. The left side is the starting point, and the right side is the end point of video image recording. Since the recording frame rate was 30 fps, 30 pixels in the horizontal direction is equivalent to 1 s in time. From the RGB intensity plot for the point on the glossy white paper, color (or hue) and intensity value of illumination from the LED ring light can be traced, analyzed, and reproduced if necessary. Any time-dependent illumination condition can be recorded and analyzed for record keeping and for future usage. Based on the time-dependent colorimetric recording and analysis results, the same illumination condition can be reproduced as needed without relying on unclear and vague human memory and rather subjective personal impressions/feelings. It is obvious that visual inspection for pH reading under certain lighting conditions can easily be misled as seen from the lower part of Figure 12b. Appearance of the same color chart is strongly influenced by hue value or color temperature of the illumination source. It is important to maintain the same lighting conditions for quantitative characterization of colors.

**Figure 13:**
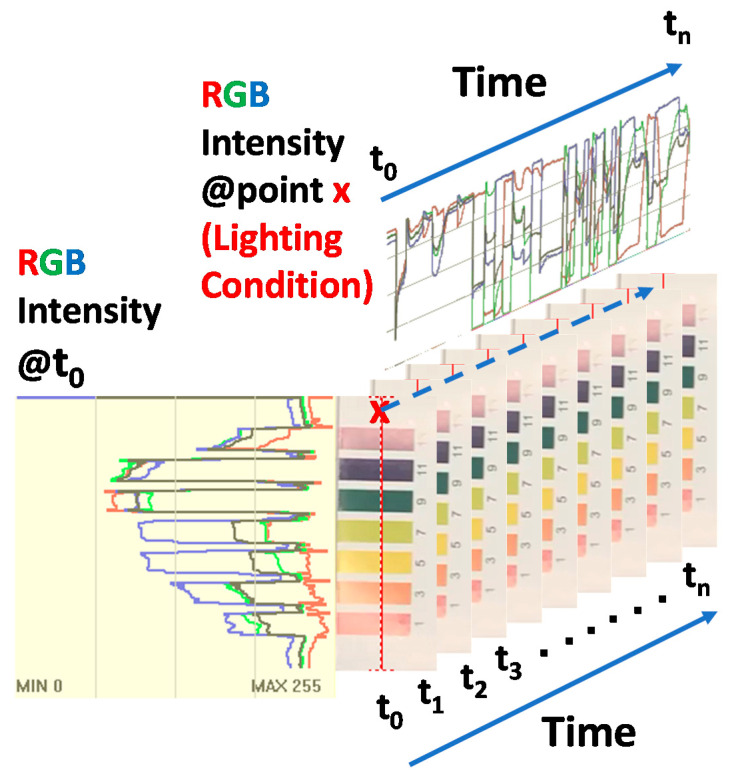
Schematic illustration of RGB channel intensity along a selected line at a given time and RGB channel intensity at a point along the time axis. (Lighting conditions are varied.)

A video image, a group of time series still images, can be dissected many different ways. A video image can be considered colorimetric information with 3D data structure (i.e., x, y, and t cube). Figure 13 shows a line cross-section on single image at a time t_0_ and a time cross-section of a point on a series of still images. Any subsets of video images such as point(s), line(s), area(s) at any particular frame(s) or entire frames can be a source of colorimetric information. Any pixel on any frame carries RGB intensity information with coordinate (relative position) and time information. Easy access to the colorimetric information on any point(s), line(s), area(s) at any particular time or interval(s) of interest becomes very important for effective and efficient use of information towards applications of interest.

Colorimetric quantification and dynamic analysis of various properties of matter, including chemical properties such as pH value, can be done through image sensor arrays. Wise use of image sensors is necessary, as machine vision will allow advances in many different fields [12,13,14,15,16,17,18]. The image information can be used as inputs for machine learning, internet of things (IoT), and artificial intelligence (AI). Development of user friendly, flexible, yet powerful, image processing/analysis software is highly desired. As an initial step, PicMan has been developed as a standalone image processing/analysis software platform for a variety of applications including semiconductor, material science, MEMS, chemistry, biological, pharmaceutical, medical, cultural heritage, and emerging applications [9,10,11,14]. An understanding of physics of image sensors and development of additional functions of image sensors will broaden the field of applications [19,20]. Hyperspectral image sensing and remote sensing applications are also actively investigated [4,21]. Development of quantitative colorimetric characterization techniques using off-the-shelf image sensors, USB cameras, and smartphones will open up newer applications.

Digital era image sensors can provide quantitative colorimetric information on objects of interest with relative position (address) information and time stamps for every single pixel on the image sensor array. Effective use of image sensors and information extraction from still and/or video images are the key for the successful integration of image processing/analysis-based tools for emerging applications in machine learning, IoT, and AI. It is time for a paradigm shift for turning image sensors into position- and time-sensitive quantitative colorimetric data sources with the aid of advanced and powerful image processing/analysis software.

### 3.4. Typical Process Flow for Similar Tasks and Benefits of Using PicMan

Other than the PicMan software package developed for these tasks, no easy to use software package is available. All tasks can be done using combinations of software packages, but it is very labor intensive and time consuming. Multiple software packages have to be running simultaneously and frequent switching between software packages is necessary. This makes still and video image analysis for colorimetric and time dependence study very difficult. For automation of image analysis, application specific algorithms have to be developed. The PicMan software package was developed for addressing manual and semi-automatic image processing needs for feasibility studies, research, and development. It can also be used in full automation mode for batch processing and industrial application with minimal customization.

We have used raw data (RGB intensity data) from each pixel for image analysis and synthesis to avoid risk of adopting inappropriate algorithms or artificial manipulation mistakes. We intended to demonstrate concepts of colorimetric quantitative analysis and time dependence analysis using the raw data from still/video image files (Videos S1, S2 and S3). Potential hardware-related issues such as different cameras/sensors, quality of the hardware, and settings (e.g., resolution, source of light/lighting conditions, etc.) can be optimized after initial tests. In most cases, cameras/sensors can be selected based on specifications (e.g., resolution, sensitivity, auto focusing, auto contrast, auto brightness control, auto exposure, file format, etc.). Light source and lighting can either be selected or designed to be suitable for specific applications. Noise reduction, smoothing, threshold switching, resolution enhancement, color adjustment, and customized functions can be added. Wireless communication for acquisition of images and analysis results through Bluetooth were successfully demonstrated.

Quantitative analysis, records, and communication eliminate uncertainties and potential miscommunication due to qualitative description. Quantitative analysis is objective, while qualitative analysis is subjective. The benefits of verbal, descriptive analysis should not be underestimated. However, it is highly desirable to use quantitative analysis whenever it is possible.

## 4. Summary

Among the five human senses, vision can provide vast amounts of information in our daily life. We naturally gather visual images and process them to extract useful information. However, our memory has limitations and our ability to communicate the information with others is also limited. With the help of image sensors, we can complement our shortfalls in memory and communication. Still images and video images acquired using image sensors are very valuable sources of information. From still images, position-sensitive, quantitative colorimetric information can be obtained. Time-dependent quantitative colorimetric information can be obtained from video images. With aid of novel image processing/analysis software, extraction of position- and time-sensitive quantitative colorimetric information was demonstrated from both still and video images. We can turn image sensors into position- and time-sensitive quantitative colorimetric data sources.

As an application example for colorimetric quantification and dynamic analysis of chemical properties, pH determination at a given point, pH variations within an area, and diffusion characteristics of liquid in litmus test paper strips are measured from still (Video S1) and video images (Videos S2 and S3) using novel image processing software. Continuous color scale generation through interpolation, using discrete reference colors, is promising for systematic conversion of colors into numerical values of interest. Feasibility of machine vision through image sensors for smart and practical quantification of visual image data has been successfully demonstrated in terms of pH measurement and diffusion speed.

A video image, a group of time series still images, can be dissected many different ways: a line cross-section on a single image at time t_0_ and a time cross-section of a point on a series of still images (Videos S2 and S3). Any subset of video images such as point(s), line(s), area(s) at any particular frame(s) or entire frames can be used as a source of colorimetric information. All pixels have RGB intensity information with coordinate (relative position) and time information. Easy access to the colorimetric information on all or desired subsets of image sensors becomes very important for effective and efficient use of information towards applications of interest. It is time to turn image sensors into position- and time-sensitive quantitative colorimetric data sources with the aid of image processing

## Supplementary Materials

The following are available online at https://www.mdpi.com/1424-8220/20/22/6418/s1, Video S1: Colorimetric data conversion from still image, Video S2: Video analysis, Video S3: Effect of illumination on color chart.
